# Antimicrobial‐Resistant Bacteria in Environmental Samples From a Rural District Focused on Large‐Scale Agricultural Production

**DOI:** 10.1111/tmi.70033

**Published:** 2025-10-01

**Authors:** Davi Abreu Carvalho Mothé, Adryene Nunes Castro, Mariah Zanon Novaes, Francisco Ozório Bessa‐Neto, Rodrigo Cayô da Silva, Creuza Rachel Vicente, Glauciomar Buss, Sarah Santos Gonçalves, Kênia Valéria dos Santos

**Affiliations:** ^1^ Biology of Microorganisms and Antimicrobials Laboratory (BiomaLab), Department of Pathology, Health Sciences Center Federal University of Espírito Santo (UFES) Vitória ES Brazil; ^2^ Laboratory of Environmental Antimicrobial Resistance (LEARN), Department of Biological Sciences (DCB), Institute of Environmental, Chemical, and Pharmaceutical Sciences (ICAQF) Federal University of São Paulo (UNIFESP) Diadema SP Brazil; ^3^ Department of Public Health Federal University of Espírito Santo (UFES) Vitória ES Brazil; ^4^ School Frederico Boldt Department of Education of Espírito Santo State Santa Maria de Jetibá Brazil; ^5^ Medical Mycology Research Center (CIMM), Department of Pathology, Health Sciences Center Federal University of Espírito Santo (UFES) Vitória ES Brazil

**Keywords:** antimicrobials, environmental contamination, Gram‐negative bacilli, livestock, multidrug‐resistant, One Health

## Abstract

Antimicrobial resistance is a growing concern, especially in regions with intense agricultural production. This study investigates the presence of antimicrobial‐resistant bacteria in Caramuru, Espírito Santo state, a rural district focused on large‐scale agricultural production in Brazil. Samples of water, soil, animal faeces and environmental surfaces were analysed using culture‐based methods, revealing the presence of multidrug‐resistant strains in agricultural and livestock environments, where antimicrobial use is common. Several bacterial species were detected, with a predominance of *Escherichia coli* , *Enterobacter* spp. and *Acinetobacter* spp., with 58.5% of the samples being resistant to at least one antimicrobial tested. The highest resistance rates among Gram‐negative bacilli were ampicillin (80%), followed by cefuroxime (70%) and ceftriaxone (55%). In addition, resistance to polymyxin B was found in 14% of the GNB isolates, including *Enterobacter asburiae* , *Enterobacter cloacae* , *E. coli* , *Acinetobacter baumannii* and *Pseudomonas aeruginosa* . The production of extended‐spectrum β‐lactamases was detected in six multidrug‐resistant *E. coli* samples isolated from river water, dog faeces and pigsty floors, while the production of metallo‐β‐lactamases was observed in *E. asburiae* from water samples from the river and toilet, as well as *E. cloacae* from coffee grounds. The *bla*_TEM‐_like gene was identified in multidrug‐resistant *E. coli* strains isolated from all the Caramuru River water and the pigsty floor samples, while *bla*_CTX‐M‐1/2_‐like was found in an *Enterobacter bugandensis* and *E. asburiae* strains isolated from flies in the toilet, respectively. These findings indicate the presence of extended‐spectrum β‐lactamase genes in different environmental and animal‐associated sources within the study area. The overlap of these detections with agricultural and surface water sites underscores the importance of monitoring antimicrobial resistance in diverse environmental compartments.

## 1 Introduction

Antimicrobial resistance (AMR) is a global public health challenge. It is estimated that 4.71 million people died in 2021 as a result of AMR, including 1.14 million deaths directly attributable to it. Projections suggest that this number will increase [1, 2].

AMR results from complex and dynamic genetic processes intensified by the continuous use of antimicrobials. The transmission routes of antimicrobial resistance genes (ARGs) are multiple and often unpredictable [3, 4]. The use of antimicrobials in human medicine, veterinary medicine, agriculture and animal husbandry brings AMR into the context of One Health. Addressing AMR in One Health perspective is even more relevant in developing countries with deficiencies in sanitation and in regulating the use and disposal of antimicrobials [5, 6].

Antimicrobials, their metabolites, pesticides and disinfectants are routinely released into the environment from industrial, agricultural and healthcare sources. Consequently, multidrug‐resistant (MDR) pathogens have been isolated from rivers [7], estuaries [8], agricultural soils [9] and other environmental matrices [10, 11] Recent studies conducted in the five regions of Brazil showed a high frequency of MDR Gram‐negative bacilli (GNB) in faeces from humans and farm animals [12, 13], food [14], water and soil [15]. Metagenomic analysis of these samples indicated the presence of 405 ARGs to 12 classes of antimicrobials, some not yet detected in Brazilian clinical strains [12, 13]. The spread of ARGs to last‐choice antimicrobials in treated sewage from urban centres in Brazil has also been demonstrated [16].

Antimicrobials in animal production and pesticides in agriculture contribute to increasing food production to meet population demand [17, 18]. However, these practices pose significant risks for the emergence of AMR in the environment and jeopardise the United Nations' sustainable development goals [19].

In Latin America, regulations on the use of antimicrobials in animal production have progressed to meet the standards set by international human and animal health authorities [20, 21]. However, there are still no specific regulations for wastewater disposal. Each producer manages waste based on local conditions, choosing to install wastewater treatment plants or dispose of it directly into the environment [22].

The largest producer of vegetables and chickens in Espírito Santo state, Brazil, is the municipality of Santa Maria de Jetibá, located in the Atlantic Forest region. It is currently the leading producer of chicken in the state and the second producer of eggs in Brazil. The production is distributed to big Brazilian centres in the Southeastern and Northeastern regions. To sustain high agricultural productivity, the region relies heavily on pesticides [23, 24], which, along with intensive farming practices [25], may contribute to environmental contamination and influence microbial resistance dynamics.

With more than 13 million chickens raised in confined systems [26], the genetic uniformity of this population and the intensive use of antimicrobials represent a considerable risk of spreading resistant pathogens into the environment [27]. To investigate AMR in this Brazilian geographic area, we designed this study in a district of Santa Maria de Jetibá, considering the precept of local intervention with a focus on global results. We chose the Caramuru district because it contributes significantly to egg production at a national level, presenting itself as a potential focus for the emergence of MDR pathogens. Given the district's similarity to other rural communities that are economically dependent on the animal production industry, the results of this study could serve as a basis for guiding political and socio‐economic actions in territories with similar characteristics.

Therefore, our objective was to characterise antimicrobial‐resistant bacteria in the rural district of Caramuru, with a particular focus on identifying carbapenemase‐ and extended‐spectrum β‐lactamase (ESβL)‐producing GNB strains.

## 2 Materials and Methods

### 2.1 Sampling Sites

**FIGURE 1:**
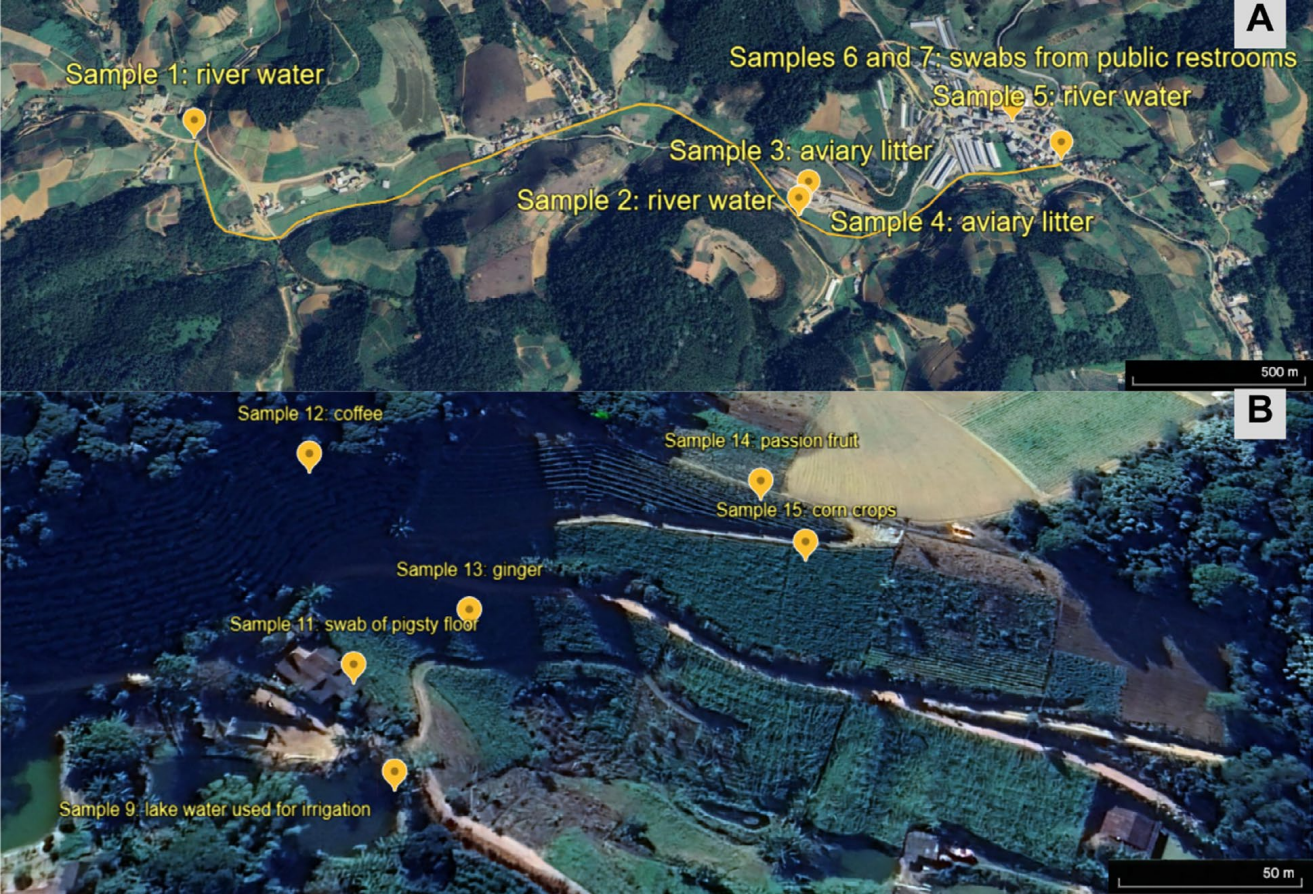
Sample collection points (yellow markers) in the Caramuru district located in the municipality of Santa Maria de Jetibá, Brazil. (A) Route of the Caramuru River with the markings of the collection points in yellow: Sample 1—river water 2.3 km upstream from the farms (20°07′09″ S 40°40′41″ W); Sample 2—river water 0.5 km upstream from the farms (20°06′10″ S 40°40′26″ W); Sample 3—poultry litter on the river bank (20°06′10″ S 40°40′26″ W); Sample 4—poultry litter in the storage shed (20°06′10″ S 40°40′26″ W); Sample 5—river water at the district's exit, 0.4 km downstream from the farms (20°05′43″ S 40°40′30″ W); Samples 6 and 7—public toilets (20°05′47″ S 40°40′35″ W); Sample 8—houseflies on the public highway (20°05′47″ S 40°40′35″ W). (B) Private rural property located 2 km from the farms (20°04′44″ S 40°41′10″ W) where they were collected: Sample 9—water from the lake used for irrigation; Sample 10—dog faeces recently deposited on the ground; Sample 11—pig faeces deposited on the floor of the pigsty; Samples 12–15—soils from coffee, ginger, passion fruit and corn crops, respectively.

Sample collection occurred in June and October 2023 in the district of Caramuru, located 16 km from the municipal seat of Santa Maria de Jetibá, Espírito Santo state, Brazil. The small urban centre of Caramuru comprises a single central street, bifurcated at its beginning and end, with shops, a school, houses and approximately 22 farm sheds (Figure 1A). The only existing urban service is the electricity supply. The landscape comprises small cultivated areas, large deforested areas on the slopes and residual Atlantic Rainforest on the hilltops. There is an intense movement of trucks transporting agricultural produce and poultry litter sold as fertiliser [28].

Water samples were collected from the Caramuru River downstream and upstream of the farms and poultry litter near the farm sheds (Figure 1A). On a family farm located 2 km from the farms mentioned above, we collected soil samples from around the region's main crops (coffee, ginger, passion fruit and corn), water samples from the lake used for irrigation, domestic dog faeces deposited on the ground and pig faeces deposited on the floor of the pigsty (Figure 1B). In the area around the farms, we collected swabs from the wet parts of the public toilets at the only gas station in the village, specimens of flies on the public highway and water from the drinking fountain at the school (Figure 1A).

### 2.2 Collection Method

Water samples were collected in 1 L sterile bottles with screw caps, while soil samples (50 g) were collected at a depth of 10 cm, approximately 15 cm from the cultivar's stem, using a sterile shovel and placed in sterile plastic bags. Additionally, poultry litter samples (50 g) were taken using sterile shovels at two different points near the farm sheds. Faecal samples and toilet surfaces were collected using Stuart swabs (Zhejiang Gongdong Medical, Zhejiang, China). Housefly specimens were captured in flight using an entomological net and sterile glass vials.

### 2.3 Detection of Thermotolerant Coliforms

The most probable number (MPN) of thermotolerant coliforms in the water samples was determined using the multiple tube technique. Three series of three tubes with 10 mL of EC (*Escherichia coli*) broth were used per sample. The first series received 10 mL of the sample and EC broth at double strength (10°); the second received 1 mL of the sample and single‐strength broth (10^−1^); and the third received 0.1 mL of the sample (10^−2^), also with single‐strength broth. We read the MPN according to the recommendations in appendix 2 of the Bacteriological Analytical Manual (BAM) [29].

### 2.4 Processing Samples to Screen for Resistant Bacteria

Initially, 250 mL of each water sample was transferred to the vacuum filtration support with a 0.45 μm membrane (Merck Millipore, USA) in an aseptic environment. After filtration, the membrane was transferred to a polypropylene tube containing 20 mL of 1% peptone water and incubated at 37°C for 4 h.

For the solid samples (crop soils and poultry litter), 25 g were diluted in 225 mL of 1% peptone water. The swabs were inserted into polypropylene tubes containing 20 mL of peptone water and vortexed for 1 min. Ten fly specimens were vortexed in 20 mL of peptone water. All the suspensions obtained were pre‐incubated at 37°C for 4 h.

Subsequently, the samples were vortexed for 1 min, and 100 μL were added to four microtubes containing 900 μL of Trypticase Soy Broth—TSB (Oxoid, Hampshire, England) plus the following selective antimicrobials (Sigma Aldrich, St. Louis, USA): (i) ceftriaxone (2 μg/mL) + vancomycin (4 μg/mL) and, (ii) ceftazidime (2 μg/mL) + vancomycin (4 μg/mL) for the selection of ESβL producing GNB; (iii) meropenem (2 μg/mL) + vancomycin (4 μg/mL), to select carbapenemase producing GNB; and (iv) meropenem (2 μg/mL) alone to select Gram‐positive cocci. After 24 h of incubation at 37°C, an aliquot of 10 μL from each microtube with turbidity was seeded onto CHROMagar Orientation Medium (BD, Maryland, USA). After incubating the CHROMagar plates at 37°C for 24 h, each colony with a distinct appearance (morphotype and pigmentation) was subcultured onto a CHROMagar plate and subsequently onto MacConkey agar (HiMedia, Mumbai, India) or Brain Heart Infusion—BHI agar (Kasvi, Curitiba, Brazil) until a pure culture was obtained [12, 13].

### 2.5 Bacterial Identification

Each isolated colony was identified at the species level using a matrix‐assisted laser desorption/ionisation with time‐of‐flight mass spectrometry (MALDI‐TOF MS) [30]. Gram staining, lactose fermentation and oxidase activity tests were also carried out for GNB. For Gram‐positive bacteria, catalase and oxidase tests were carried out to identify and exclude possible inconsistencies.

### 2.6 Antimicrobial Susceptibility Testing

The disc‐diffusion technique was used for all bacterial isolates with cut‐off points defined according to the Brazilian Committee on Antimicrobial Susceptibility (BrCAST)/European Committee on Antimicrobial Susceptibility Testing (EUCAST) guidelines (eucast.org), as detailed in Tables 2 and 3. The following antimicrobial discs (Laborclin, Brazil) were tested for GNB: ampicillin (10 μg), amoxicillin/clavulanate (20/10 μg), piperacillin/tazobactam (30/6 μg), cefuroxime (30 μg), cefoxitin (30 μg), cefepime (30 μg), ceftazidime (10 μg), ceftriaxone (30 μg), aztreonam (30 μg), ertapenem (10 μg), imipenem (10 μg), meropenem (10 μg), ciprofloxacin (5 μg), levofloxacin (5 μg), gentamicin (10 μg), tobramycin (10 μg) and sulfamethoxazole/trimethoprim (23.75/1.25 μg). For Gram‐positive bacteria, ampicillin (10 μg), cefoxitin (30 μg), imipenem (10 μg), ciprofloxacin (5 μg), levofloxacin (5 μg), linezolid (10 μg), tetracycline (30 μg) and rifampicin (5 μg) were tested.

For polymyxin B and vancomycin (Sigma Aldrich, St. Louis, USA), the minimum inhibitory concentration (MIC) was determined by cation‐adjusted Mueller Hinton broth (Difco, BD, Franklin Lakes, USA) microdilution technique according to the BrCAST/EUCAST protocol (eucast.org). *E. coli* ATCC 25922, *Pseudomonas aeruginosa* ATCC 27853 and *Enterococcus faecalis* ATCC 29212 were used as quality controls. The bacteria were classified in terms of their MDR profile according to the criteria of Magiorakos et al. [31], which define MDR as acquired non‐susceptibility to at least one agent in three or more antimicrobial categories.

### 2.7 Phenotypic Detection of ESBL and Carbapenemase Production

The β‐lactamase phenotypic detection test was carried out on all GNB isolates resistant to 3rd generation cephalosporins and/or carbapenems. Initially, a bacterial suspension with a turbidity equivalent to the 0.5 standard on the McFarland scale was prepared in sterile 0.85% saline and spread on Mueller‐Hinton agar (Difco, BD, Franklin Lakes, USA).

Carbapenemase production was detected by the inhibitory activity of 0.1 M EDTA (Bio‐Rad, California, USA) and phenylboronic acid (AFB) (Sigma Aldrich, St. Louis, USA) (40 mg/mL). Discs of imipenem (IMI) (10 μg), meropenem (MER) (10 μg) and blank disc (Laborclin, Brazil), with and without EDTA (0.1 M) or AFB (40 mg/mL), were added to the Mueller‐Hinton agar plates previously inoculated with the test GNB isolates. After incubating the plates at 37°C for 24 h, the diameter of the halos formed was read and interpreted. Isolates with a difference in diameter of ≥ 5 mm for IMI + EDTA and MER + AFB compared to IMI and MER alone were considered to be producers of carbapenemase of class B (metallo‐β‐lactamase—MβL) and of class A, respectively [32].

To detect ESβL production, the disc approximation test was carried out, which is based on adding a disc of amoxicillin/clavulanic acid (20/10 μg) to the centre of the Mueller‐Hinton agar plate previously inoculated with the bacterial suspension and discs of cefepime (30 μg), ceftriaxone (30 μg), ceftazidime (10 μg) and aztreonam (30 μg) disposed at a distance of 20–25 mm from it. The test was positive when the inhibition zone of the disc was enlarged towards the amoxicillin/clavulanic acid disc, forming a ghost zone [32, 33].

### 2.8 Antimicrobial Resistance Genes Detection

The ARGs were screened for 37 GNB isolates that showed resistance to at least one antimicrobial. Total bacterial DNA was extracted using 5% Chelex 100 (Sigma Aldrich, St. Louis, USA). Negative extraction controls (no‐template extractions processed alongside samples) were included in each batch to monitor potential contamination during the extraction process. Conventional qualitative PCR was carried out, according to the antimicrobial susceptibility profile, to search for ARGs to β‐lactams (*bla*_SHV‐_like, *bla*_TEM_‐like, *bla*_CTX‐M‐1/2_‐like, *bla*_CTX‐M‐14_‐like, *bla*_OXA23_‐like, *bla*_OXA‐24/40_‐like, *bla*_OXA‐51_‐like, *bla*_OXA‐58_‐like, *bla*_OXA‐143_‐like, *bla*_IMP_‐like, *bla*_VIM_‐like, *bla*_SIM_‐like, *bla*_GIM_‐like, *bla*_SPM_‐like and *bla*_KPC_‐like), fluoroquinolones (*qnrA*, *qnrB*, *qnrC*, *qnrD* and *qnrS*), aminoglycosides (*armA*, *npmA*, *rmtA*, *rmtB*, *rmtC*, *rmtD*, *rmtE*, *rmtF* and *rmtG*) and for polymyxins (*mcr‐1* to *mcr*‐*5*). The specific primers and amplification conditions are described in the Supporting Information. Positive controls were not available for all *bla*_IMP_‐like, *bla*_VIM_‐like, *bla*_SIM_‐like, *bla*_GIM_‐like, *bla*_SPM_‐like, *bla*_KPC_‐like and *mcr*‐1 to *mcr‐5*; therefore, these results should be interpreted with caution. Negative controls were included in all runs.

PCR products were separated by electrophoresis on 1% agarose gel, stained with ethidium bromide, with both positive and negative gel controls included in assays to verify amplification success and rule out contamination. A 100 bp DNA ladder was used in all gels as a molecular size marker to confirm the expected amplicon length, while positive gel controls consisted of PCR products from reference strains and negative gel controls consisted of reactions without template DNA. The gels were then analysed using ImageQuant LAS 4000 (GE Healthcare, Little Chalfont, UK).

### 2.9 Statistical Analysis

Chi‐square and Fisher's exact tests were utilised to compare the MDR rate between samples from poultry farms and those from a rural property using GraphPad Prism software, version 9.3.0.

## 3 Results

### 3.1 Testing for Thermotolerant Coliforms

All water samples from the Caramuru River and rural property were contaminated with thermotolerant coliforms, except for the water from the school drinking fountain. The highest numbers were found 0.5 km upstream from the farms and at the district's exit (Table 1).

**TABLE 1:** Number of positive tubes and interpretative results for the MPN of thermotolerant coliforms in the water samples collected from the Caramuru River, the rural property's lake and the school drinking fountain.

Spot	No. of positive tubes	MPN/mL	CI
Sample 1: river water	3–3–1	460	90–2000
Sample 2: river water	3–3–2	1100	180–4100
Sample 5: river water	3–3–3	> 1100	420 to —
Sample 9: lake water used for irrigation	3–2–0	98	18–120
Drinking fountain at school	0–0–0	< 3	0–9.5

Abbreviations: CI, confidence interval 95%; MPN, most probable number.

### 3.2 Bacterial Profile

**FIGURE 2:**
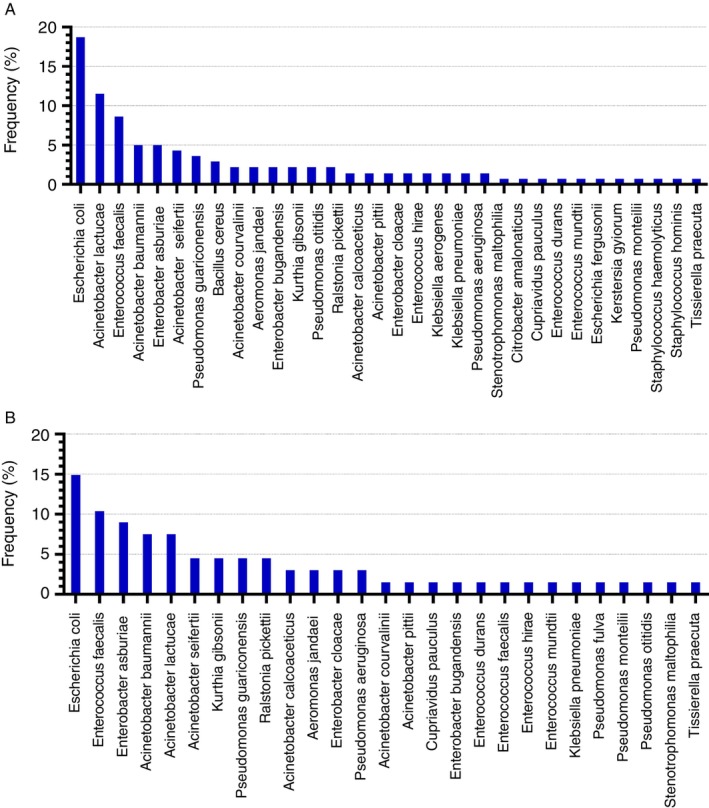
Overview of bacterial species (%) identified by MALDI‐TOF MS isolated in the rural district of Caramuru, Brazil. (A) Initial screening (*n* = 163). (B) After exclusion of replicates (*n* = 67).

A total of 163 isolates were obtained in the selective pressure conditions, among which 138 were identified at the species level by MALDI‐TOF MS (Figure 2). The only negative sample for resistant bacteria isolation came from the school drinking fountain water. A significant diversity of species was observed in the samples, with the highest abundance recorded for *E. coli* (18.7%), followed by *Acinetobacter lactucae* (11.5%) and *Enterococcus faecalis* (8.6%) (Figure 2A). Species recorded at the same sampling point were counted only once. After excluding probable replicate samples, the final number of isolates were reduced to 67 (Figure 2B), of which 20 (29.8%) are classified within the *Enterobacterales* order. Non‐fermenting GNB accounted for 37.3%, comprised of the genera *Acinetobacter* (*n* = 17), *Pseudomonas* (*n* = 8), *Stenotrophomonas* (*n* = 1) and *Ralstonia* (*n* = 3) (Figure 2B). In relation to the Gram‐positive cocci, 11 *Enterococcus* spp. isolates were recovered, with a predominance of *E. faecium* (*n* = 7). The other environmental species identified were *Aeromonas* spp. (*n* = 2), *Kurthia* spp. (*n* = 3), *Tissierella* sp. (*n* = 1) and *Cupriavidus* sp. (*n* = 1) (Figure 2B).

### 3.3 Antimicrobial Susceptibility Profile

**FIGURE 3:**
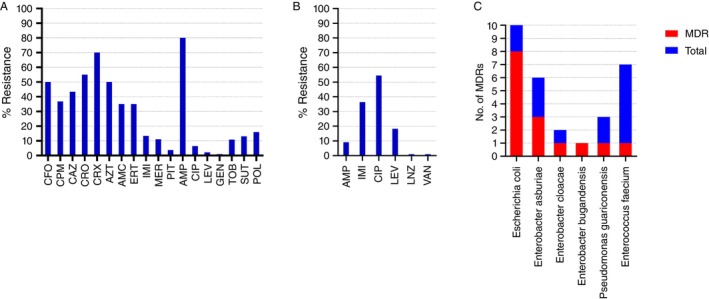
Frequency of resistant bacteria per antimicrobial agent tested. (A) Gram‐negative bacilli (*n* = 43). (B) Gram‐positive cocci (*n* = 11). (C) Distribution of MDR bacteria by species. AMC, amoxicillin/clavulanate; AMP, ampicillin; AZT, aztreonam; CAZ, ceftazidime; CFO, cefoxitin; CIP, ciprofloxacin; CPM, cefepime; CRO, ceftriaxone; CRX, cefuroxime; ERT, ertapenem; GEN, gentamicin; IMI, imipenem; LEV, levofloxacin; MDR, multidrug‐resistant; MER, meropenem; PIT, piperacillin/tazobactam; POL, polymyxin; SUT, sulfamethoxazole/trimethoprim; TOB, tobramycin.

Antimicrobial susceptibility tests were carried out for all isolates, except *Ralstonia pickettii* (*n* = 3), *Kurthia gibsonii* (*n* = 3), *Tissierella praecuta* (*n* = 1) and *Cupriavidus pauculus* (*n* = 1), which do not have breakpoints. Resistance to at least one antimicrobial was observed in 42.4% (*n* = 25/59) of all samples. Susceptibility with increased exposure (former intermediate) to at least one antimicrobial was detected in 64.4% (*n* = 38/59) of isolates. The antimicrobials with the highest resistance rates among GNB isolates were ampicillin (80%), followed by cefuroxime (70%) and ceftriaxone (55%). Among the carbapenems, 35%, 13% and 11% of isolates were resistant to ertapenem, imipenem and meropenem, respectively (Figure 3A). Resistance to polymyxin was verified in 14% (*n* = 7/48) of the GNB isolates: *E. asburiae* (*n* = 3) isolated from river water, toilet and poultry litter (MIC ranging between 4 and 64 mg/L); *E. cloacae* (*n* = 1) recovered from poultry litter (MIC = 4 mg/L); *E. coli* (*n* = 1) isolated from the floor of the pigsty (MIC = 4 mg/L); *Acinetobacter baumannii* (*n* = 1) isolated from coffee‐growing soil (MIC = 4 mg/L); and *P. aeruginosa* (*n* = 1) isolated from passion fruit‐growing soil (MIC = 8 mg/L) (Table 2). Among the Gram‐positive bacteria, the highest resistance rates were observed for ciprofloxacin (54%) and imipenem (36%) (Figure 3B).

**TABLE 2:** Microbiological data of 48 Gram‐negative bacteria isolates recovered in the Caramuru district according to the collection site with their respective collection sites, species identification, growth inhibition halos (mm), interpretative category and beta‐lactamase production.

Sites	Code	Species	CFO	CPM	CAZ	CRO	CRX	AZT	AMC	ERT	IMI	MER	PIT	AMP	CIP	LEV	GEN	TOB	SUT	POL^a^	ESBL	MBL	Genes
Sample 1: river water	A4	*Klebsiella pneumoniae*	26	28	26	25	24	33	25	30	26	26	27	14	33	32	21	23	26	2			—
	A5	*Pseudomonas guariconensis*		24	23			16			29	26			33	25				2			—
	A6	*Aeromonas jandaei*		33	32			41							37	35			34				—
	A7	*Enterobacter asburiae*	9	31	28	28	22	24	8	8	7	6	28	6	35	33	23	22	32	2	−	+	—
	C1	*Enterobacter asburiae*	6	35	21	22	6	28	6	27	28	33	28	6	28	24	22	21	28	64			—
	C2	*Acinetobacter lactucae*	11	20	18	19		17	16	—	30	25			28	27	23	25		2			—
	**C3**	***Escherichia coli***	**21**	**11**	**14**	**6**	**6**	**10**	**12**	**28**	**27**	**28**	**26**	**6**	**24**	**20**	**21**	**20**	**30**	**2**	**+**	**−**	***blaTEM***
	C5	*Acinetobacter baumannii*	14	24	23	21		20	22	—	32	30			32	31	24	24		2			—
Sample 2: river water	A23	*Pseudomonas otitidis*		28	27			28			22	17	26		35	28				2			—
	A24	*Escherichia coli*	25	30	24	30	21	33	25	33	27	31	27	19	37	34	21	20	29	2			—
	**A25**	***Pseudomonas fulva***		**20**	**19**			**13**			**11**	**20**	**20**		**27**	**18**				**1**			**—**
	**C9**	***Escherichia coli***	**23**	**6**	**6**	**6**	**6**	**8**	**13**	**25**	**12**	**28**	**22**	**6**	**6**	**7**	**22**	**10**	**6**	**2**	**+**	**−**	***blaTEM***
	C10	*Acinetobacter seifertii*	12	22	17	17		16	21		34	28			28	26	24	25		2			—
	C12	*Acinetobacter baumannii*	10	20	19	17		14	20		33	30			28	28	26	26		2			—
Sample 3: aviary litter	**C38**	***Enterobacter asburiae***	**6**	**26**	**18**	**20**	**6**	**19**	**7**	**24**	**24**	**28**	**27**	**6**	**35**	**33**	**21**	**21**	**30**	**4**	**−**	**−**	**—**
	C39	*Pseudomonas aeruginosa*		28	26			29			28	37	30		33	24				2			—
	C40	*Acinetobacter lactucae*	20	28	26	21		25	19		18	15			43	41	34	31		1			—
Sample 4: aviary litter	C31	*Acinetobacter pittii*	10	20	19	18		16	15		34	32			27	26	20	27		2			—
	**C33**	***Escherichia coli***	**26**	**13**	**17**	**7**	**6**	**16**	**23**	**29**	**28**	**31**	**28**	**6**	**22**	**23**	**20**	**15**		**2**	**−**	**−**	**—**
	**C36**	***Enterobacter cloacae***	**6**	**34**	**18**	**23**	**6**	**23**	**7**	**24**	**25**	**30**	**28**	**6**	**35**	**33**	**23**	**21**	**32**	**4**	**−**	**−**	**—**
Sample 5: river water	A45	*Enterobacter asburiae*	9	32	26	29	23	35	9	33	27	33	31	6	37	35	25	24	30	2			—
	A46	*Pseudomonas monteilii*		23	19			15			30	26	25		29	24				2			—
	C23	*Acinetobacter baumannii*	10	21	22	20		14	17		32	28			30	29	23	21		2			—
	**C24**	***Escherichia coli***	**24**	**6**	**6**	**6**	**6**	**6**	**17**	**29**	**30**	**31**	**26**	**6**	**35**	**30**	**21**	**21**	**6**	**2**	**+**	**−**	***blaTEM***
Sample 6: swab from women's public restroom	**C85**	***Enterobacter asburiae***	**6**	**24**	**22**	**15**	**6**	**32**	**9**	**6**	**6**	**6**	**25**	**6**	**31**	**34**	**21**	**12**	**38**	**4**	**−**	**+**	**—**
Sample 7: swab from men's public restroom	**C89**	***Enterobacter asburiae***	**6**	**29**	**14**	**22**	**6**	**27**	**8**	**21**	**23**	**27**	**27**	**6**	**32**	**29**	**20**	**22**	**27**	**2**			***blaCTX1/2***
Sample 8: flies on public roads	C46	*Acinetobacter courvalinii*	19	20	18	21		20	20		36	29			29	29	24	20		2			—
	**C48**	***Enterobacter bugandensis***	**6**	**25**	**6**	**10**	**6**	**14**	**6**	**20**	**23**	**27**	**17**	**6**	**31**	**30**	**25**	**22**	**25**	**2**	**−**	**−**	***blaCTX1/2***
Sample 9: lake water used for irrigation	**A33**	***Escherichia coli***	**13**	**6**	**6**	**15**	**6**	**6**	**33**	**14**	**28**	**18**	**26**	**20**	**28**	**30**	**19**	**6**	**31**		−	−	—
	**C18**	***Escherichia coli***	**25**	**10**	**14**	**6**	**6**	**11**	**15**	**31**	**29**	**33**	**27**	**6**	**35**	**34**	**21**	**20**	**6**	2	**+**	**−**	***blaTEM***
	C54	*Acinetobacter lactucae*	13	19	19	20		20	20		35	31			32	30	25	24		2			—
Sample 10: swab of dog faeces	**C42**	***Escherichia coli***	**25**	**6**	**13**	**6**	**6**	**8**	**22**	**32**	**31**	**32**	**27**	**6**	**35**	**30**	**20**	**20**	**31**	**2**	**+**	**−**	**−**
Sample 11: swab of pigsty floor	**C76**	***Escherichia coli***	**19**	**12**	**11**	**12**	**6**	**14**	**16**	**31**	**32**	**32**	**25**	**6**	**23**	**21**	**19**	**19**	**17**	**4**	**+**	**−**	***blaTEM***
	C79	*Acinetobacter lactucae*	10	21	19	20		20	18		41	40			32	29	26	30		1			—
	C80	*Acinetobacter baumannii*	8	17	18	19		18	16		22	18			31	30	23	26		1			—
Sample 12: soil of coffee	A66	*Acinetobacter lactucae*	11	15	17	16		16	16		34	32			28	29	26	26		2			—
	A68	*Enterobacter cloacae*	10	28	21	29	23	24	8	8	16	10	31	6	37	34	25	25	32	2	−	+	—
	A70	*Aeromonas jandaei*		36	32			40							37	35			30		−	−	—
	C92	*Acinetobacter calcoaceticus*	7	16	17	19		16	15		30	32			33	32	29	28		2			—
	C93	*Acinetobacter baumannii*	10	18	20	17		20	18		32	29			29	28	21	22		4			—
Sample 13: soil of ginger	**A77**	***Pseudomonas guariconensis***		**6**	**6**			**6**			**29**	**23**	**22**		**17**	**21**				**2**	−	−	—
	C99	*Acinetobacter seifertii*	8	17	15	17		14	15		28	29			31	30	25	27		2			—
	C103	*Stenotrophomonas maltophilia*																	33				—
Sample 14: soil of passion fruit	A88	*Pseudomonas aeruginosa*		23	24			25			29	27	23		25	21				8			—
	A90	*Pseudomonas guariconensis*		20	20			17			29	25	21		30	20				2			—
	A89	*Escherichia coli*	29	29	28	31	23	26	25	28	25	26	30	20	36	33	22	21	30	2			—
	C108	*Acinetobacter seifertii*	7	15	17	16		16	17		30	28			33	32	25	26		2			—
Sample 15: soil of corn crops	C105	*Acinetobacter calcoaceticus*	10	18	17	19		14	15		30	30			30	29	22	25		2			—

*Note*: 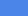 Intrinsic resistance; 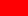 Resistance; 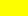 Susceptible, increased exposure. Bold represents MDR bacteria. (+) positive; (−) negative.

Abbreviations: AMC, amoxicillin/clavulanate; AMP, ampicillin; AZT, aztreonam; CAZ, ceftazidime; CFO, cefoxitin; CIP, ciprofloxacin; CPM, cefepime; CRO, ceftriaxone; CRX, cefuroxime; ERT, ertapenem; GEN, gentamicin; IMI, imipenem; LEV, levofloxacin; MER, meropenem; PIT, piperacillin/tazobactam; POL, polymyxin B; SUT, sulfamethoxazole/trimethoprim; TOB, tobramycin.

^a^ For polymyxin B, the broth microdilution method was used, so the number refers to the MIC (mg/L).

Species with MDR phenotype were found in all water samples from the Caramuru River ( *E. coli* and *Pseudomonas fulva* ), poultry litter ( *E. asburiae* , *E. cloacae* and *E. coli* ), toilet swab ( *E. asburiae* ), flies (*E. bugandensis*), in lake water ( *E. coli* ), in domestic dog faeces ( *E. coli* ), on the floor of the pigsty ( *E. coli* ) and in ginger‐growing soil (*P. guariconensis*) (Table 2). The frequency of MDR isolates was 25.4% (15/59), the majority corresponding to *E. coli* (*n* = 8) and *E. asburiae* (*n* = 3) isolates. Among the MDR *E. coli* strains, strain C9 (river water) showed resistance to 3rd generation cephalosporins, aztreonam, imipenem and fluoroquinolones; strain A33 (lake water) presented resistance to ertapenem and meropenem and strain C76 (floor of the pigsty) was resistant to polymyxin B (Table 2). Among the MDR *E. asburiae* , strain C38 (poultry litter) showed resistance to polymyxin B, while strain C85 presented resistance to polymyxin B and carbapenems (Table 2). Among the Gram‐positive strains, the MDR phenotype was detected in only one strain of *Enterococcus faecium* recovered from the toilet (Table 3).

**TABLE 3:** Microbiological data of 11 Gram‐positive bacteria isolated in Caramuru district according to the collection sites, species identification, growth inhibition halos (mm) and interpretative category.

Sites	Code	Species	AMP	IMI	CIP	LEV	LNZ	VAN^a^
Sample 1: river water	C8	*Enterococcus faecium*	26	22	6	22	29	1
Sample 2: river water	A19	*Enterococcus faecium*	23	24	20	19	24	1
	C17	*Enterococcus faecium*	24	8	6	17	23	1
Sample 5: river water	C25	*Enterococcus faecium*	23	15	15	16	26	1
Sample 6: swab from women's public restroom	C87	*Enterococcus hirae*	22	20	21	20	20	1
Sample 7: swab from men's public restroom	C90	*Enterococcus durans*	25	29	22	22	20	1
	**C98**	***Enterococcus faecium***	**6**	**9**	**9**	**10**	**20**	**1**
Sample 9: lake water used for irrigation	C61	*Enterococcus faecalis*	27	27	10	20	20	2
	C66	*Enterococcus mundtii*	25	22	19	20	21	1
Sample 10: swab of dog faeces	C45	*Enterococcus faecium*	26	22	6	15	22	1
Sample 11: swab of pigsty floor	C81	*Enterococcus faecium*	24	23	13	14	23	2

*Note*: 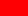 Resistance; 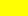 Susceptible, increased exposure. Bold represents MDR bacteria.

Abbreviations: AMP, ampicillin; CIP, ciprofloxacin; IMI, imipenem; LEV, levofloxacin; LNZ, linezolid; VAN, vancomycin.

^a^ The broth microdilution method was used for Vancomycin, so the number refers to the MIC (mg/L).

Analysing the samples from the areas where the farms are concentrated and from the rural property separately, we observed a higher frequency of GNB resistance in the former. Resistance to at least one antimicrobial was observed in 57% (*n* = 16/28) of the isolates where the farms are concentrated and 35% (7/20) in the samples collected from the rural property. A similar pattern was found in the analysis of MDR bacteria, with a frequency of 35% (*n* = 10/28) in the farm region and 25% (*n* = 5/20) in the rural property. However, this difference between the regions was not statistically significant in the Chi‐square (*p* = 0.0606) and Fisher's exact (*p* = 0.1541).

### 3.4 β‐Lactamase Phenotypic Detection

ESβL production was detected in six MDR *E. coli* isolates recovered from river water samples (C3, C9 and C24), lake water (C18), dog faeces (C42) and pigsty floors (C76). MβL production was detected in two *E. asburiae* isolates recovered from river water (A7) and toilet water (C85) and in one *E. cloacae* isolate recovered from coffee‐growing soil (A68). None of the isolates was positive in the phenotypic test for the production of Class A carbapenemases (Table 2).

### 3.5 Antimicrobial Resistance Genes Screening

Of the 14 ARGs investigated, two were detected in the study samples: *bla*_TEM_‐like and *bla*_CTX‐M‐1/2_‐like. The *bla*_TEM_‐like gene was identified in five MDR *E. coli* isolates recovered from the river water samples (C3, C9, C18 and C24) and from the floor of the pigsty (C76). In addition, the *bla*_CTX‐M‐1/2_‐like gene was detected in one *E. bugandensis* isolate (C48) recovered from flies and in one *E. asburiae* isolate (C89) recovered from the surface of the toilet bowl (Table 2).

## 4 Discussion

In the present study, thermotolerant coliforms were detected at multiple points along the Caramuru River. Although resistant bacterial isolates were detected more frequently in samples collected near the farms, some isolates were also present at more distant sites. These spatial patterns indicate a non‐uniform distribution of antimicrobial‐resistant isolates across this rural watershed, with higher frequencies near agricultural areas.

Poultry litter is waste from poultry production, which, when improperly stored in open sheds or dumped into the environment during transportation, can reach streams and rivers through rainwater. In this study, we detected MDR bacteria ( *E. cloacae* , *E. coli* and *E. asburiae* ) at poultry litter collection points and in the river water downstream from the farms. *E. coli* resistant to various antimicrobials has already been reported in poultry litter [34], but our study is the first to report *Enterobacter* species resistant to cephalosporins in this type of sample in Brazil. In addition, we report the isolation of polymyxin‐resistant bacteria and acarbapenem‐resistant *Acinetobacter* isolate, highlighting the role of farm waste in disseminating MDR bacteria and their genes into the environment.

The *E. coli* strains isolated from all the river sites exhibited similar AMR profiles. This similarity indicates that comparable resistance patterns are present along the river's course, although the genetic relatedness of the isolates was not determined. These findings align with studies conducted in rural regions showing that agricultural management practices can select for resistant bacteria in the environment [35]. All the *E. coli* isolated from the water were ESBL producers, representing a potential risk of introducing resistant bacteria and ARGs into downstream water treatment systems and into agricultural irrigation, with possible impacts on food safety and public health [36].

Enterobacteria with similar susceptibility profiles were identified in samples of domestic dog faeces and the floor of the pigsty. Given their proximity to the pond used for crop irrigation, these animals may have contact with this water, which could represent one of several possible exposure routes; however, the actual direction or source of bacterial dissemination was not determined. Previous studies have documented the role of domestic and farm animals in spreading resistant pathogens in the environment and indicate that resistance in *Enterobacterales* could potentially threaten human health [13, 37].

The bacteria isolated from water samples and the floor of the pigsty carried the *bla*_TEM_‐like gene, a β‐lactamase determinant commonly associated with mobile genetic elements [38, 39, 40]. While the presence of *bla*_TEM_‐like in both aquatic and livestock environments does not prove direct transfer, it highlights that these settings share reservoirs of critical ARGs. Indeed, the widespread detection of *bla*_TEM_‐like and related genes such as *bla*_CTX‐M_‐like in diverse environmental matrices underscores the importance of continued surveillance to track their distribution among bacterial populations relevant to human and animal health [7]. The *bla*_CTX‐M‐1/2_‐like gene was detected in the fly samples and on the surface of the men's toilet. Previous studies have described flies' role in spreading ESβL‐encoding genes, such as *bla*_CTX‐M‐15_ [41]. Detecting the *bla*_CTX‐M‐1/2_‐like gene in our samples reinforces that flies can transport resistant microorganisms between different locations, including domestic surfaces, organic waste and communal environments. In our study, the detection of *bla*_CTX‐M‐1/2_‐like in flies and on surfaces in collective use, such as toilets, illustrates the various routes by which MDR bacteria and ARGs can spread [41].

The *Enterobacter* spp. stood out in our study due to: (i) resistance to carbapenems and positive tests for MβL production in *E. asburiae* isolated from distinct environments (Caramuru River, women's public restroom, coffee‐growing soil); (ii) resistance to polymyxin B in *E. asburiae* (Caramuru River, poultry litter, women's public restroom); and (iii) resistance to 3rd generation cephalosporins in isolates found in poultry litter and public restroom. *Enterobacter* species have already been found in environmental samples of water [42], soil and sewage [43], mainly carrying the genes *bla*_*VIM*_‐like and *bla*_*IMP*_‐like.

The *P. guariconensis* identified in this study has previously been associated with plantation soils [44]. Although the cephalosporin and monobactam resistance observed in the soil sample has not previously been reported in environmental isolates, it is in line with previously described clinical findings, which reported *P. guariconensis* resistant to these antimicrobials in a case of asymptomatic bacteriuria [45]. This species has already been associated with rare infections such as necrotising fasciitis [46], reinforcing its importance for future studies.

The genus *Acinetobacter*, especially the *A. calcoaceticus‐baumannii* complex, which includes the most clinically relevant species, was frequent in the present study. One of the significant challenges related to this genus is its intrinsic resistance to various antimicrobials used in clinical practice, which restricts therapeutic options, mainly when resistance to carbapenems occurs [47, 48]. In our study, a single sample of *A. lactucae* isolated from poultry litter showed resistance to carbapenems. On the other hand, the resistance to polymyxin B found in a sample of *A. baumannii* isolated from coffee‐growing soil was unexpected and deserves further characterisation in the future since, in Brazil, the AMR rates of this genus to polymyxins are usually low [49], around 20% during 2018 and 2021 [50].

Polymyxin B resistance, observed in seven GNB isolates in the present study, is worrying since this antimicrobial is one of the last therapeutic options for severe MDR infections, especially those resistant to carbapenems [48]. The polymyxin B‐resistant isolates included *E. asburiae* isolates recovered from distinct sources (water, poultry litter, women's public restroom), *E. coli* from pigsty floor, *A. baumannii* from coffee soil and *P. aeruginosa* from passion fruit soil. Most of these isolates were classified as MDR. Although uncommon, resistance to polymyxins has already been reported in *Enterobacter* spp. and *E. coli* isolated from water [51] in pig faeces [52], respectively. Resistance to polymyxins in environmental bacteria (water and soil samples) is often associated with the plasmid‐mediated gene *mcr* [53, 54, 55]. In our study, phenotypic resistance to polymyxins was observed, yet all resistant samples tested negative for mcr 1–5. Since a positive control for the PCR assay was not included, these negative results should be interpreted with caution. Moreover, the observed resistance may also be mediated by alternative genetic determinants or epigenetic regulatory mechanisms beyond the mcr genes.

Among the Gram‐positive bacteria, only the genus *Enterococcus* was detected at all four water collection points on the Caramuru River, reinforcing the evidence of faecal contamination in the environment [56]. The main problem associated with species such as *E. faecium* and *E. faecalis* is resistance to vancomycin [57, 58]. In our study, all *Enterococcus* spp. isolates were susceptible to vancomycin, but resistant to ciprofloxacin (54%) and imipenem (36%) was observed, which is worrying as these antimicrobials are used to treat severe infections.

Certain antimicrobials are still permitted in Brazil as performance‐enhancing additives in poultry production [59]. In the context of Santa Maria de Jetibá, one of the country's largest egg producers, these substances can favour the selection of resistant bacteria in the environment, facilitating their spread. However, more research is needed to assess the real impact of this scenario on antimicrobial resistance, including studies with more extensive collections and continuous monitoring.

Our results indicate the interconnection between the environments studied and the emerging challenges related to AMR within the One Health framework. We identified MDR GNB, including strains resistant to last‐line agents such as polymyxins and carbapenems, in multiple environmental samples. These findings occurred in areas where intensive chicken and egg production activities take place, and the observed patterns warrant further investigation to clarify potential environmental drivers and impacts.

Detecting resistant *Enterobacter* and ESBL‐producing *E. coli* isolates in river water, agricultural soil, poultry litter and flies highlights the relationship between human practices (agriculture, animal husbandry and waste management) and the spread of AMR in the environment. Previous work has documented the exchange of these resistance determinants between animals, humans and environmental compartments [60], underscoring the need for integrated surveillance across One Health domains.

In addition, the diversity of samples containing MDR bacteria, from domestic dog faeces to soil from food crops, reinforces that the problem of AMR has several interfaces, covering human, animal and environmental health in an interconnected way. The rural environment, which often combines intensive agricultural practices and proximity to natural areas, was especially relevant in this study, serving as a hotspot for the emergence of MDR strains.

Our study is constrained by a limited number of samples and by the random sub‐selection of enriched isolates specifically aimed at recovering resistant strains—inclusion of all isolates (including sensitive ones) would have generated an unmanageable volume of samples and analyses. Access challenges to animal production areas and rural properties further restricted sampling. Nevertheless, given the region's geography and proximity to the Caramuru River, these findings still provide valuable insights into the patterns of selective pressure and distribution of antimicrobial resistance in this setting.

## 5 Conclusion

MDR GNB ( *E. coli* , *Enterobacter* spp. and *Pseudomonas* spp.) with resistance to last‐line clinical antimicrobials were detected in all the environmental samples collected in the region of the farms and less frequently in the agricultural soil of the rural property of Caramuru district. Poultry litter proved to be an important reservoir of resistant bacteria, where we isolated for the first time *E. cloacae* and *E. asburiae* resistant to 3rd‐generation cephalosporins and polymyxin B and imipenem‐resistant *A. lactucae*. These findings underscore the importance of a One Health approach that prioritises environmental genomic surveillance and proper on‐farm waste management to mitigate the spread of AMR in agroecosystems with similar characteristics.

## Conflicts of Interest

The authors declare no conflicts of interest.

## Supporting information


**Data S1:** tmi70033‐sup‐0001‐supinfo.pdf.
