# Supplementary material for: Antimicrobial‐Resistant Bacteria in Environmental Samples From a Rural District Focused on Large‐Scale Agricultural Production
Source: Trop Med Int Health. 2025 Oct 1;30(12):1269–82. doi: 10.1111/tmi.70033 (PMC12675321; doi:10.1111/tmi.70033)
Supplement: Supplementary file 1 — Data S1: tmi70033‐sup‐0001‐supinfo.pdf. [file TMI-30-1269-s001.pdf]

Primer	Sequence	Amplicon size (bp)	Initial denaturation	Cycles	Temperature and time			Final extension	Reference
					Denaturation	Annealing	Extension		
blaSHV-F	CTTGACCGCTGGGAAACGG	200	95ºC for 10min	35	95ºC for 30s	52ºC for 50s	72ºC for 60s	72ºC for 10min	NICOLETTI, A.G. Caracterização genética e bioquímica da BKC-1, uma nova carbapenemase da classe A de Ambler, isolada de amostras clínicas de Klebsiella pneumoniae. Tese de doutorado. UNIFESP, São Paulo, 145pp., 2014
blaSHV-R	AGCACGGAGCGGATCAACGG								
blaTEM-F	CCCTTATTCCCTTTYTTGCGG	650	95ºC for 10min	35	95ºC for 30s	52ºC for 50s	72ºC for 60s	72ºC for 10min	
blaTEM-R	AACCAGCCAGCCWGAAGG								
blaCTX-M-1/2-F	ATGTGCAGYACCAGTAA	512	94ºC for 10min	35	94ºC for 20s	58ºC for 45s	72ºC for 60s	72ºC for 10min	
blaCTX-M-1/2-R	CGCTGCCGGTTTTATCSCCC								
blaCTX-M-14-F	GCACGATGACATTCGGG	874	94ºC for 10min	35	94ºC for 20s	58ºC for 45s	72ºC for 60s	72ºC for 10min	
blaCTX-M-14-R	AACCCACGATGTGGGTAGC								
blaOXA23-F	GATCGGATTGGAGAACCAGA	500	95ºC for 10min	30	95ºC for 30s	52ºC for 40s	72ºC for 50s	72ºC for 10min	WOODFORD N, ELLINGTON MJ, COELHO JM, TURTON JF, WARD ME, BROWN S, AMYES SG, LIVERMORE DM. Multiplex PCR for genes encoding prevalent OXA carbapenemases in Acinetobacter spp. Int J Antimicrob Agents. 2006 Apr;27(4):351-3.
blaOXA23-R	ATTTCTGACCGCATTTCCAT								
blaOXA24/40-F	GGTTAGTTGGCCCCCTTAAA	246	95ºC for 10min	30	95ºC for 30s	52ºC for 40s	72ºC for 50s	72ºC for 10min	
blaOXA24/40-R	AGTTGAGCGAAAAGGGGATT								
blaOXA51-F	TAATGCTTTGATCGGCCTTG	353	95ºC for 10min	30	95ºC for 30s	52ºC for 40s	72ºC for 50s	72ºC for 10min	
blaOXA51-R	TGGATTGCACTTCATCTTGG								
blaOXA58-F	AAGTATTGGGGCTTGTGCTG	599	95ºC for 10min	30	95ºC for 30s	52ºC for 40s	72ºC for 50s	72ºC for 10min	
blaOXA58-R	CCCCTCTGCGCTCTACATAC								
blaIMP-F	GAATAGRRTGGCTTAAYTCTC	188	94ºC for 10min	30	94ºC for 30s	58ºC for 50s	72ºC for 30s	72ºC for 10min	MENDES RE, KIYOTA KA, MONTEIRO J, CASTANHEIRA M, ANDRADE SS, GALES AC, PIGNATARI AC, TUFIK S. Rapid detection and identification of metallo-beta-lactamase-encoding genes by multiplex real-time PCR assay and melt curve analysis. J Clin Microbiol. 2007 Feb;45(2):544-7.
blaIMP-R	CCAAACYACTASGTTATC								
blaVIM-F	GTTTGGTCGCATATCGCAAC	382	94ºC for 10min	30	94ºC for 30s	58ºC for 50s	72ºC for 30s	72ºC for 10min	
blaVIM-R	AATGCGCAGCACCAGGATAG								
blaSIM-F	GTACAAGGGATTCGGCATCG	589	94ºC for 10min	30	94ºC for 30s	58ºC for 50s	72ºC for 30s	72ºC for 10min	
blaSIM-R	TGGCCTGTTCCCATGTGAG								
blaSPM-F	CTAAATCGAGAGCCCTGCTTG	798	94ºC for 10min	30	94ºC for 30s	53ºC for 40s	72ºC for 30s	72ºC for 10min	
blaSPM-R	CCTTTTCCGCGACCTTGATC								
blaKPC-F	TCGCTAAACTCGAACAGG	860	94ºC for 10min	35	94ºC for 30s	55ºC for 30s	72ºC for 50s	72ºC for 10min	Poirel L, Walsh TR, Cuvillier V, Nordmann P. Multiplex PCR for detection of acquired carbapenemase genes. Diagn Microbiol Infect Dis. 2011 May;70(1):119-23. doi: 10.1016/j.diagmicrobio.2010.12.002. Epub 2011 Mar 12. PMID: 21398074.
blaKPC-R	TTACTGCCCGTTGACGCCCAATCC								
qnrA-F	AGAGGATTTCTCACGCCAGG	580	95ºC for 10min	25	95ºC for 45s	58ºC for 45s	72ºC for 15s	72ºC for 10min	CATTOIR V, POIREL L, ROTIMI V, SOUSSY CJ, NORDMANN P. Multiplex PCR for detection of plasmid-mediated quinolone resistance qnr genes in ESBL-producing enterobacterial isolates. J Antimicrob Chemother. 2007 Aug;60(2):394-7.
qnrA-R	TGCCAGGCACAGATCTTGAC								
qnrB-F	GGMATHGAAATTCGCCACTG	264	95ºC for 10min	25	95ºC for 45s	58ºC for 45s	72ºC for 15s	72ºC for 10min	
qnrB-R	TTTGCYGYYCGCCAGTCGAA								
qnrS-F	GCAAGTTCATTGAACAGGGT	428	95ºC for 10min	25	95ºC for 45s	58ºC for 45s	72ºC for 15s	72ºC for 10min	
qnrS-R	TCTAAACCGTCGAGTTCGGCG								
qnrC-F	GCGAATTTCCAAGGGGCAAA	135	95ºC for 10min	25	95ºC for 45s	58ºC for 45s	72ºC for 15s	72ºC for 10min	KRAYCHETE GB, BOTELHO LA, CAMPANA EH, PICÃO RC, BONELLI RR. Updated Multiplex PCR for Detection of All Six Plasmid-Mediated qnr Gene Families. Antimicrob Agents Chemother. 2016;60(12):7524-7526. Published 2016 Nov 21.
qnrC-R	ACCCGTAATGTAAGCAGAGCAA								
qnrD-F	AGGTGTAGCATGTATGGAAAAGC	691	95ºC for 10min	25	95ºC for 45s	58ºC for 45s	72ºC for 15s	72ºC for 10min	
qnrD-R	ACATTGGGGCATTAGGCGTT								
armA-F	ATTCTGCCTATCCTAATTGG	316	94ºC for 5min	35	94ºC for 20s	60ºC for 60s	72ºC for 75s	72ºC for 10min	
armA-R	ACCTATACTTTATCGTCGTC								
npmA-F	GGAGGGCTATCTAATGTGGT	386	94ºC for 5min	35	94ºC for 20s	60ºC for 60s	72ºC for 75s	72ºC for 10min	
npmA-R	GCCCAAAGAGAATTAAACTG								
rmtA-F	CTAGCGTCCATCCTTTCCTC	762	94ºC for 5min	35	94ºC for 20s	60ºC for 60s	72ºC for 75s	72ºC for 10min	
rmtA-R	TTGCTTCCATGCCCTTGCC								
rmtB-F	GAATGGGGGCGGCATAAATC	652	94ºC for 5min	35	94ºC for 20s	60ºC for 60s	72ºC for 75s	72ºC for 10min	

rmtB-R	AAGTTCTGTTCCGATGGTCTT	652	94ºC for 5min	35	94ºC for 20s	60ºC for 60s	72ºC for 75s	72ºC for 10min	
rmtC-F	CGACGTGTAACTGAGAGGCTT	500	94ºC for 5min	35	94ºC for 20s	60ºC for 60s	72ºC for 75s	72ºC for 10min	CASSU-CORSI D, MARTINS WM, NICOLETTI AG, ALMEIDA LG, VASCONCELOS AT, GALES AC. Characterisation of plasmid-mediated rmtB-1 in Enterobacteriaceae clinical isolates from São Paulo, Brazil. Mem Inst Oswaldo Cruz. 2018;113 (12):e180392.
rmtC-R	TCGCCTGACGGATCGGATAA								
rmtD-F	GACCGAGCGCGAATACAAAC	440	94ºC for 5min	35	94ºC for 20s	60ºC for 60s	72ºC for 75s	72ºC for 10min	
rmtD-R	CGGAAACGATGCGACGACGAT								
rmtE-F	GGAGGGCTATCTAATGTGGT	355	94ºC for 5min	35	94ºC for 20s	60ºC for 60s	72ºC for 75s	72ºC for 10min	
rmtE-R	GCCCAAAGAGAATTAAACTG								
rmtF-F	ATTCTGCCTATCCTAATTGG	434	94ºC for 5min	35	94ºC for 20s	60ºC for 60s	72ºC for 75s	72ºC for 10min	
rmtF-R	ACCTATACTTTATCGTCGTC								
rmtG-F	CTAGCGTCCATCCTTTCCTC	535	94ºC for 10min	35	94ºC for 20s	60ºC for 60s	72ºC for 75s	72ºC for 10min	
rmtG-R	TTGCTTCCATGCCCTTGCC								
mcr 1-F	AGTCCGTTTGTTCTTGTGGC	320	94ºC for 15min	25	94ºC for 30s	58ºC for 90s	72ºC for 60s	72ºC for 10min	
mcr 1-R	AGATCCTTGGTCTCGGCTTG								
mcr 2-F	CAAGTGTGTTGGTCGCAGTT	715	94ºC for 15min	25	94ºC for 30s	58ºC for 90s	72ºC for 60s	72ºC for 10min	REBELO, A. R. et al. Multiplex PCR for detection of plasmid-mediated colistin resistance determinants, mcr-1, mcr-2, mcr-3, mcr-4 and mcr-5 for surveillance purposes. Euro Surveillance, v. 23, n. 6, p. 17-00672, fev. 2018. DOI: 10.2807/1560-7917.ES.2018.23.6.17-00672. Erratum in: Euro Surveillance, v. 23, n. 7, fev. 2018. DOI: 10.2807/1560-7917.ES. 2018.23.7.180215-1.
mcr 2-R	TCTAGCCCGACAAGCATACC								
mcr 3-F	AAATAAAAATTGTTCCGCTTATG	929	94ºC for 15min	25	94ºC for 30s	58ºC for 90s	72ºC for 60s	72ºC for 10min	
mcr 3-R	AATGGAGATCCCCGTTTTT								
mcr 4-F	TCACTTTCATCACTGCGTTG	1116	94ºC for 15min	25	94ºC for 30s	58ºC for 90s	72ºC for 60s	72ºC for 10min	
mcr 4-R	TTGGTCCATGACTACCAATG								
mcr 5-F	ATGCGGTTGTCTGCATTTATC	1644	94ºC for 15min	25	94ºC for 30s	58ºC for 90s	72ºC for 60s	72ºC for 10min	BOROWIAK, M. et al. Identification of a novel transposon-associated phosphoethanolamine transferase gene, mcr-5, conferring colistin resistance in d-tartrate fermenting Salmonella enterica subsp. enterica serovar Paratyphi B. Journal of Antimicrobial Chemotherapy, v. 72, n. 12, p. 3317-3324, 2017. DOI: 10.1093/jac/dkx327.
mcr 5-R	TCATTGTGGTTGTCCTTTTCTG								
ERIC 1	ATGTAAGCTCCTGGGGATTCAC	-	95ºC for 7min	26	95ºC for 30s	48ºC for 30s	72ºC for 120s	72ºC for 10min	VERSALOVIC, J.; KOEUTH, T.; LUPSKI, R. Distribution of repetitive DNA sequences in eubacteria and application to finerpriting of bacterial enomes. Nucleic Acids Research, v. 19, n. 24, p. 6823–6831, 1991.
ERIC 2	AAGTAAGTGACTGGGGTGAGCG								
